# Host–Pathogen Interactions: A Biological *Rendez-Vous* of the Infectious Nonself and Danger Models?

**DOI:** 10.1371/journal.ppat.0020044

**Published:** 2006-05-26

**Authors:** Jean-Nicolas Tournier, Anne Quesnel-Hellmann

*“Les phénomènes biologiques présentent une telle complexité que lorsqu'on a établi une règle les concernant, il faut toujours s'attendre à des exceptions plus ou moins nombreuses. [Biological phenomena are so complex, that when a new rule has been established, it is expected to find several exceptions.]” —Elie Metchnikov [1]*

## 

Until recently, immunologists had thought of the immune system as a complex cellular web aimed at protecting the body against marauding microbes, by producing a highly specialized, specific, and adaptive response. This adaptive response involves the production of a specific receptor for each antigenic motif (e.g., B cell receptors, antibodies, and T cell receptors), using specific mechanisms of recombination and rearrangements of somatic genes. Inherent molecular complexity of the mechanisms supporting the regulated production of such receptors monopolized the attention of most immunologists for several decades. In the late 80s, Janeway explored the “road not taken” and reversed the previous dogma of adaptive immunity supremacy, stressing a more prominent role for innate immunity (a first line of defense based on the recognition of conserved pathogen motifs) [2]. In a seminal paper, he was the first to imagine new rules for the immune system to account for “the immunologist's dirty little secret,” which had downplayed the need for bacterially derived adjuvants to efficiently induce adaptive immune responses [3]. Janeway proposed that the immune system recognizes a conserved molecular pattern, called pathogen-associated molecular pattern (PAMP), displayed by pathogens but not normally found on host cells (e.g., lipopolysaccharide, peptidoglycans, nonmethylated CpG, and double-stranded RNA). He predicted “nonclonally distributed” (e.g., germline encoded) receptors for PAMP, and coined the term “pattern recognition receptors” (PRRs), which he identified a first type in 1997 as the Toll-like receptors (TLRs) [4]. The discovery of the TLRs led to the emergence of a revolutionary and fertile field now studied by many. Finally, Janeway proposed that the innate immune system distinguished “infectious nonself” from “noninfectious self” [5,6]. The infectious nonself (INS) model revolutionized immunology, although it left several important questions unanswered. For example, why do PRRs not discriminate between nonpathogenic and pathogenic microbes [6]? This question has been partly answered by recent data revealing a novel function of TLRs in controlling intestinal epithelial homeostasis through interactions with nonpathogenic agents [7], while another effort identified a “combinatorial security code” by which dendritic cells (DCs) discriminate between pathogens [8]. A second major question regarding the INS model concerns activation of the immune system in nonpathogen-associated situations such as allograft rejection or autoimmunity. For autoimmunity, recent works demonstrate a crucial role of TLR activation in at least two murine models of autoimmune diseases such as type 1 diabetes and lupus [9,10].

In the early 90s, Polly Matzinger proposed the “danger” model as an alternative and comprehensive view of the immune system, where endogenous danger signals (e.g., cytokines) released from infected cells could affect the function of antigen-presenting cells (APCs) without directly exposing APCs to PAMPs [11]. The danger model had broader implications than the INS model, since it could predict the activation of the immune system in various situations such as anti-infectious and antitumor immunity, autoimmunity, or allograft rejection. Experimental data have been raised subsequently showing that necrotic but not apoptotic cells trigger the activation of DCs, which represent crucial APCs coupling innate and adaptive responses [12,13]. The danger model, however, faced several objections raised by the INS model. Why did evolution select a complex PRR system if endogenous danger sensors would be sufficient, and what are the relevant signals (exogenous or endogenous) for mounting an efficient immune response? Why should the immune system be cognizant of tissue damage that doesn't involve microbes, and would the immune system be activated to induce autoreactive responses following major cellular distress?

Recently, Seong and Matzinger stressed that the semantic distinction between exogenous and endogenous danger signals is artificial, since most PAMP such as lipopolysaccharide and bacterial DNA are not displayed by “healthy” pathogens but, instead, are released after significant pathogen stress [14]. PAMP and endogenous danger signals could each be considered damage signals released either by the host or by the pathogen. PRR such as TLR recognize either PAMP or endogenous signals, whereas at least one danger receptor senses PAMP. To account for this, Seong and Matzinger proposed a synthetic model based upon the recognition of damage-associated molecular pattern (DAMP) by DCs. These damage patterns might be associated with a hydrophobic molecular region exposed after stress and subsequently recognized as a universal danger signal.

Here, we explore the DAMP model through a broader host–pathogen perspective. We propose that pathogens encode DAMP-interfering molecules that we suggest could be termed “negative signals” (NS). These NSs might be molecules displayed or encoded by a pathogen that are not recognized as DAMP by PRR, and block or modify the intracellular signaling downstream of PRRs to induce tolerance. We identified two major NS strategies used by microbes to overcome the host immune system: first, interfering directly with APC's PRR; or second, triggering apoptosis of phagocytes that in turn inhibit proximal APCs (Figure 1). Clearly, pathogens use strategies that impair PRR as well as danger receptors. We focus here on studies that illustrate the main mechanisms used by bacteria and viruses to escape host DAMP recognition.

**Figure 1:**
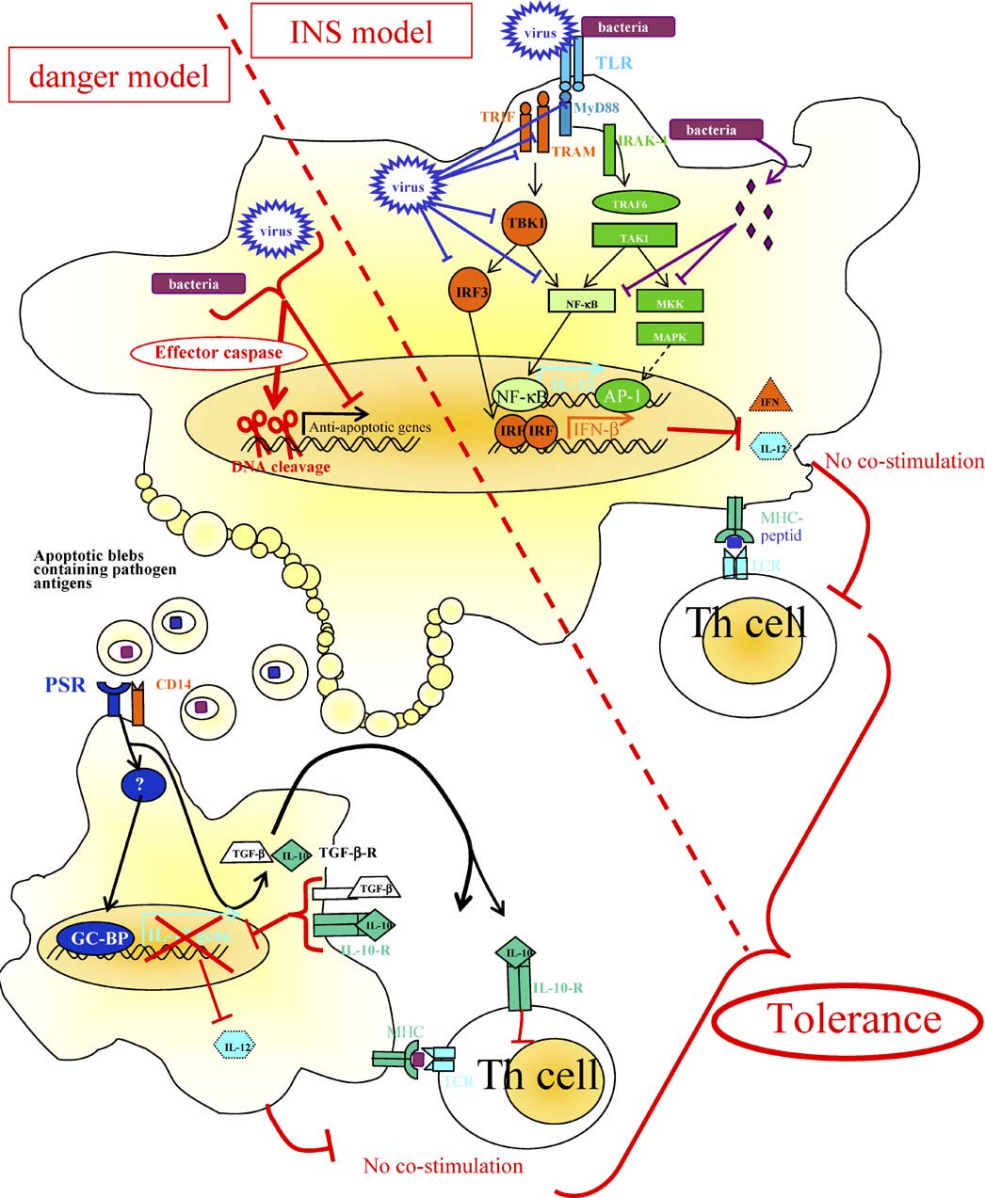
Pathogens Improve Their Survival by Overcoming INS Immune Defenses When Targeting TLRs or “Danger” Immune Defenses When Targeting Apoptosis Microbes undertake two major strategies to overcome the host immune system: interfering directly with APC's pattern recognition receptors (INS model side), or triggering apoptosis of phagocytes that in turn inhibits proximal APCs (danger model side). Both strategies ultimately lead to tolerance by inducing presentation of antigen in absence of crucial co-stimulatory molecules. Viruses have been shown to have a myriad of strategies to disrupt the TLR cascade, mainly focusing on the IFN response, while bacteria act far downstream on mitogen-activated kinase kinases and NF-κB. Pathogen-induced cell death induces the release of cellular blebs expressing phosphatidylserine (PS) that are rapidly internalized by neighboring cells or phagocytes. Infected, apoptotic cells may send these NSs to limit the effectiveness of antigen presentation by neighboring uninfected APCs to T helper cells. In this situation, without co-stimulatory molecules and secreted IL-12 but in presence of IL-10 and transforming growth factor β, ultimately tolerance to microbial antigens will be induced. AP1, activator protein 1; GC-BP, GC binding protein; IFN, interferon; IL, interleukin; IRAK, interleukin-1-associated kinase; IRF, IFN-regulatory factor; MAPK, mitogen-activated protein kinase; MHC, major histocompatibility complex; MKK, MAPK kinase; MyD88, myeloid differentiation primary-response factor 88; NF-κB, nuclear factor κB; PSR, phosphatidylserine receptor; TAK, TGF-β-activated kinase; TBK, TRAF-family-member-associated NF-κB activator-binding kinase; TCR, T cell receptor; TGF, transforming growth factor; TLR, Toll-like receptor; TRAF, TNF-receptor-associated factor; TRAM, TRIF-related adaptator molecule; TRIF, TIR-domain-containing adaptor inducing IFN-β

## Pathogen NS Interfering with PRR Signaling

Numerous pathogens negatively regulate DAMP signaling pathways, thus turning off the immune response [15,16]. As TLRs play a central role in pathogen recognition and signaling, and many downstream elements of the transduction cascade are now identified [17,18], we have confined our analysis to pathogen-interfering effects on TLR signaling.

The TLR pathway induces interferon (IFN) production through several signaling proteins that ultimately lead to the activation of the transcription factors NF-κB, IFN-regulatory factor (IRF)3, and IRF7. Viruses possess myriad strategies to disrupt the TLR cascade, mainly focusing on the IFN response, while bacteria tend to act further downstream on mitogen-activated protein kinases (MAPK), mitogen-activated kinase kinases, and NF-κB [15,16,19,20]. Viruses, indeed, have to block the IFN response that crucially depends on IRFs, and the connection between TLR and IRF involves the adaptor proteins immediately downstream of TLRs [21–23]. Vaccinia virus (VV) possesses the greatest diversity of NS strategies for interacting with TLR pathways. The VV A46R protein, a homolog of Toll-like-interleukin-1 receptor (TIR), targets the host TIR adaptors MyD88, as well as the MyD88-adaptor-like TIR-domain-containing adaptor-inducing IFN-β (TRIF), and TRIF-related adaptor molecule, thereby interfering with downstream activation of MAPKs, NF-κB, and IRF3 [24,25]. A52R VV protein is a nonredundant effector that associates with TNF-receptor-associated factor 6 and interleukin-1-associated kinase 2, blocking the activation of NF-κB [26]. VV further encodes N1L, a protein that associates with I-κB kinase complex and TRAF-family-member-associated NF-κB activator-binding kinase-1 for inhibition. As a result, N1L disrupts NF-κB signaling and IRF3 signaling pathways. VV also encodes E3L that inhibits IRF3 and IRF7 phosphorylation through direct interactions [27,28].

Despite high NS variety, bacteria act essentially on MAPK and NF-κB [15,16]. For example, Bacillus anthracis lethal factor (LF) is a zinc-dependent metalloprotease that cleaves six of the seven mitogen-activated kinase kinases [29], as well as impairs IRF3 [30]. As a result, we and others have shown that LF disrupts innate and adaptive immunity through DC impairment [31,32]. Yersinia pestis YopJ and *Y*. *enterocolitica* YopP effector proteins of the type III secretion system target MAPK signaling pathways and NF-κB [33]. Interestingly, YopJ and YopP belong to a group of effectors produced by other pathogens such as *Salmonella* (AvrA) [34].

## Pathogens Exploit the Anti-Inflammatory Properties of Apoptosis

Surprisingly, numerous pathogens take care to induce a silent death of host-infected cells via apoptosis [35], suggesting that this pathway confers an evolutionary advantage to the pathogen. For viruses, in particular, survival is highly dependent on their ability to take rapid control of the host cell cycle, and most of their NS strategies are based upon regulating apoptosis [36]. Here, we have confined our analysis to the mechanisms of apoptosis that are specifically regulated by pathogens in APCs (macrophages and DCs).

Viruses have developed an extensive molecular repertoire designed to disrupt DC survival, as these APCs are one of the first lines of cellular defense [37]. Not surprisingly, VV induces programmed cell death among macrophages and DCs, although the specific viral NSs remain to be characterized [38,39]. Numerous bacterial pathogens also influence apoptosis of APCs. *B*. *anthracis* induces the apoptosis of some murine macrophages through an LF-dependent inhibition of p38 [40,41]. Yersinia spp. have at least two ways to induce macrophage apoptosis: first, using TLR2 and TLR4 signaling, and involving TRIF [42]; and second, by triggering the apoptosis of macrophages through the type III secretion system effector proteins YopP and YopJ [43–45]. Besides the direct inhibitory effects of YopP and YopJ on MAPK and NF-κB pathways disrupting the transcription of antiapoptotic genes, it has been demonstrated that YopP specifically triggers the apoptotic pathways above tBid [46].

Viruses have indeed developed many capabilities to silently kill APCs and to rescue other cell types from programmed cell death. What consequences might this have for the host? Besides the direct removal of DCs as potent immunostimulatory cells, APC apoptosis represents a supplemental manner to attenuate the immune response [47]. One of the main features of apoptosis is that cytoplasmic contents are not spilled into the extracellular milieu, and that apoptotic bodies expressing phosphatidylserine blebs from apoptotic cells are rapidly internalized by neighboring cells or phagocytes. The rapid engulfment of apoptotic cells and apoptotic bodies by neighboring APCs prevents the release of potentially toxic or immunogenic intracellular contents from the dying cells. Moreover, apoptotic cells deliver active anti-inflammatory and other inhibitory signals to DCs such as IL-10 and transforming growth factor β [48–50]. A recent study further demonstrated that apoptotic cells inhibit IL-12p70 secretion by a phosphatidylserine-driven mechanism [51]. The signals delivered by apoptotic cells to the immune system have been recently invoked as a means to maintain peripheral tolerance under a steady state [52]. Further, apoptotic cells as a source of antigens for cross-presentation have indeed been shown to play an important role in maintaining peripheral tolerance in several models [53,54].

Here, we propose that during infections, control of DC apoptosis is hijacked by pathogens to “turn down” the immune response. Apoptotic blebs may constitute “negative bullets,” transmitting both NS and pathogen antigens, inhibiting the recruitment and activation of proximal APCs, and ultimately inducing tolerance. It makes biological sense that pathogens would amplify their immunosuppressive effects in this manner, since one infected apoptotic cell releasing a bulk of blebs may impair numerous APCs in the microenvironment. As a paradigm, measles virus triggers DC apoptosis and causes severe immunosuppression [55]. The triggering of APC apoptosis by pathogens could then be regarded as an efficient weapon of immune silencing. This alternative strategy may represent a potent NS for danger signals, just as efficient as NSs that disrupt TLR signaling directly.

The long history of host–pathogen coevolutionary interactions has led pathogens to develop efficient tools for impairing the host immune system. Immunologists have much to learn from these pathogen strategies, which could help us to imagine and design new potent tools to control the immune response in various immunopathological conditions such as diabetes and related autoimmune diseases, graft rejection, or allergy. Pathogens, on the other hand, have evolved under a selective immune pressure that allows them to thrive whether or not they comprehend immunologist's models.

## Abbreviations

APC: antigen-presenting cell
DAMP: damage-associated molecular pattern
DC: dendritic cell
IFN: interferon
INS: infectious nonself
IRF: IFN-regulatory factor
MAPK: mitogen-activated protein kinase
NS: negative signal
PAMP: pathogen-associated molecular pattern
PRR: pattern recognition receptor
TIR: Toll-like-interleukin-1 receptor
TLR: Toll-like receptor
TRAF: TNF-receptor-associated factor
TRIF: TIR-domain-containing adaptor inducing IFN-β
VV: Vaccinia virus
